# Analysis of a q-deformed hyperbolic short laser pulse in a multi-level atomic system

**DOI:** 10.1038/s41598-022-13407-7

**Published:** 2022-06-03

**Authors:** N. Boutabba, S. Grira, H. Eleuch

**Affiliations:** 1 Fatima College of Health Sciences, Institute of Applied Technology, Abu Dhabi, UAE; 2 Department of Applied Sciences and Mathematics, Abu Dhabi University, Abu Dhabi, UAE; 3 Department of Applied Physics and Astronomy, University of Sharjah, Sharjah, UAE; 4 Institute for Quantum Science and Engineering, Texas A&M University, College Station, 77843 TX USA

**Keywords:** Quantum physics, Nonlinear optics

## Abstract

A fast laser pulse with a q-deformed hyperbolic function shape is investigated in a Multi-level atomic system. Therefore, we first derive the exact solution of the Bloch equations describing a two-level atom excited by a q-deformed laser pulse with dephasing and time-dependent detuning. Next, we analyze the dynamic of the atomic population inversion at resonance and off-resonance of a Rubidium 87 three-level atom driven by a classical weak field and a strong q-deformed control laser. Finally, in order to get a deeper insight of the probe field’s absorption and dispersion properties, we investigate the coherence’s dependence on the q-deformation. Our work demonstrates that, the dynamic of the atomic system can be fully controlled through the manipulation of the asymmetry scaling parameter q of the q-deformed hyperbolic laser wave-form.

## Introduction

In the field of quantum optics, light matter interaction plays an important role in optoelectronic devices^1–4^, sensing^5^, infrared (IR) nano-antennas^6^, mid-infrared molecular vibrations driven by femto-second lasers^7^ as well cavity quantum electrodynamics (QED) and nano-resonnators^8,9^. Furthermore, it provides significant insights to the field of bio-photonic medical sciences based on the analysis of the near infrared light (NIR) of biological complexe structures^10^. Light absorption, scattering, fluorescence, and reflectance emitted by biochemical processes, for example, are used to diagnose and identify diseases^11^. Indeed, absorption and emission spectra typically occur on an ultra-short time scale as the electromagnetic radiation interacts with matter via excited electronic transitions on the sub-nanometer length^12^. Therefore, employing external ultrashort laser beams as an excitation source of atomic systems is a key road to achieve high resolution of charge carrier dynamics. For instance, bright and dark optical solitons with variable ultraslow group velocities were generated from a probing light by controlling the strength of a coupling light and/or the magnetic field^13^. In addition, a useful approach based on both optical switching and bistability for a two-level atom in a ring cavity and under the effect of an external magnetic field was proposed. The device exhibits optical bistability and switching abilities under an electromagnetically induced transparency regime, which may be controlled by modulating the magnetic field or the coupling light. Such technique has pertinent applications in the construction of optical switches and storage devices^14^. As a result, significant efforts in the field of quantum optics contributed to the implementation of high-precision tailored laser beams capable of trapping and micro-manipulating matter^15^.

The detection of optical scattering on particles, initially reported in 1970 by Arthur Ashkin and later awarded the Nobel Prize in 2018, was made possible by the development of powerful, highly focused laser beams^16,17^. The approach employs an ultra-short chirped pulse to generate an 11-order greater gradient force in the opposite direction of the beam, capable of trapping, holding, and moving a single particle or atom. This technique, also known as the single-beam gradient force trap or optical tweezers^16,18^, is a breakthrough in medical sciences as well as quantum information, which includes light storage, solitons trapping, fast quantum logic gates, and quantum simulations^19,20^.

These astounding discoveries and accomplishments are the consequence of combining high temporal coherence with precise resolution by using frequency comb sources in addition to customizing laser beams^21^. Nonetheless, while the quantum control of single-qubit nuclear spins and atomic hyperfine ground states is possible^22^, strong transitions control is limited by the availability of sub-nanosecond laser waveforms^23^. Hence, the field of pulse shaping is still an active research area.

Recently, Yudi et al, used various delayed picosecond pulses to generate arbitrarily shaped optical waveforms with a bandwidth of 30 GHz and a duration of 100 ps, resulting in “super-resolved” spectroscopic signals, which could open up intriguing possibilities in quantum optics based on fast laser cooling and atom interferometry with mode-locked lasers^24^. In addition, theoretical investigation of various waveforms in a four-level atomic system allowed the control of the negative refraction in a Rb 87 atomic media^25^. Besides, the introduction of a complex cosine amplitude combined with a phase jump enabled the atomic population inversion in a V-type three level system, in contrast with the standard case where the laser beam is a constant or a gaussian field^26^. Finally, rising cosine, sinc, gaussian, root rising cosine, than-hyperbolic, exponential, and their modified forms such as QEXP, PEXP, and DGF are frequently utilized in digital communications^27^. The synthesis of these programmable wave forms is based on standard processing techniques such as the direct space to time shaping, the process of combining dispersion and phase imaging, and the shaping via the spatial light modulators are discussed in Ref.^28^.

In this paper, we perform a theoretical analysis of a multilevel atom excited by a laser pulse with a q-deformed hyperbolic function shape. First, we study the dynamics of a two-level system excited by q-deformed laser beam. Next, we investigate the optical properties of a three-level atomic system interacting with two electromagnetic fields. It is a structure of a V-type configuration where 1 weak field drives the Stokes transition while the q-deformed shaped laser drives the pump transition. This paper is organized as follows: in “Exact solution of the Bloch equations for the q-deformed function”, we establish a method to solve the Bloch equations, with exact analytical solutions, in a two level atom with dephasing, chirped detuning and where the control field exciting the atom is a q-deformed hyperbolic function. The details are reported in the Supplementary Materials. In “Discussion”, we discuss the atomic population inversion as well as the real and the imaginary part of the coherence under the q-deformed waveform pumping effect. Finally the conclusions are given in “Conclusion”.

## Exact solution of the Bloch equations for the q-deformed function

Deformations in quantum systems have been extensively studied in a variety of fields, ranging from the quantum control of single atoms to the manipulation of many particles, which is based on the non-linear interactions of high peak intensity pulses (femtosecond) with atoms and molecules, and hence requires precisely tailored waveforms^29^. The q-deformation of hyperbolic potential, first proposed by Arai^30^and afterwards studied by a number of authors^31–34^, is a type of parameter scaling symmetry in a system. These functions are also used to describe systems where the symmetry is broken including among others atom-trapping potentials, statistical distributions in Bose Einstein condensates and complex vibration–rotation energy structure of multi-electron atom^35^.

In general, the Arai q-deformed function is given by:

$$\begin{aligned} cosh_q(x)=\frac{e^x+qe^{-x}}{2}\\ sech_q(x)=\frac{1}{cosh_q(x)}. \end{aligned}$$

**Figure 1:**
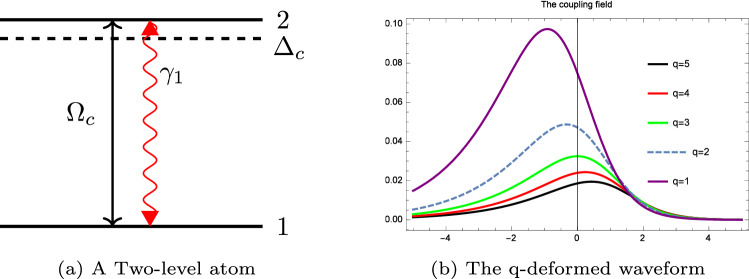
(**a**) A two-level atom excited by a strong field with Rabi-frequency $\Omega _{c}$, (**b**) The q-deformed laser shape for various q-deformations.

We now consider a two-level atom under dephasing and described by two energy eigenstates $|1\rangle$ and $|2\rangle$. In the realistic scenario, the dephasing is caused by various processes such as the inhomogeneous broadening in atomic ensembles, collisions in atomic gases, and atomic decoherences induced by the optical pumping^36^. Such two level atom configuration is illustrated in Fig. 1 where the total Hamiltonian in the interaction picture and under the rotating-wave approximation is given by:

1 $$\begin{aligned} H=\Delta (t)\sigma _{x}\frac{\hbar }{2}+\Omega (t)\sigma _{z}\frac{\hbar }{2}. \end{aligned}$$

Here, $\sigma _{x},$ and $\sigma _{z}$ represent the Pauli matrices and the Rabi frequency is written in terms of the field-atom interaction as: $\Omega (t)=-d\frac{E(t)}{\hbar }$.

The Rabi frequency exciting the two level atom is given by:

2 $$\begin{aligned} \Omega = \frac{4B e^{-3\Gamma t}}{(1+e^{-c\alpha ^2}e^{-2\Gamma t})^2} \end{aligned}$$

and the detuning $\Delta = \omega _{2}-\omega _{1}$ is considered to be time dependent:

3 $$\begin{aligned} \Delta = \frac{4A e^{-2\Gamma t}}{(1+e^{-c\alpha ^2}e^{-2\Gamma t})^2}, \end{aligned}$$

where the positive constants A and B are: $A=\frac{1}{2} \alpha ^2 e^{-c\alpha ^2} \Gamma$ and $B=\frac{1}{2} \alpha ^2 e^{\frac{-c\alpha ^2}{2}} \Gamma$. $\Gamma$ is the decoherence rate also known as the dephasing. Hence, we can write: $\Omega =Be^{-\Gamma t} sech^2_q(-2\Gamma t)$ and $\Delta =A sech^2_q(-2\Gamma t)$ It is worth noting that, the temporal dynamics of the density matrix elements are related to the atomic populations (which are the diagonal elements), and to the coherence (which is presented by the off-diagonal elements). Thus, the Bloch equations can be written as:

4 $$\begin{aligned} \begin{bmatrix} \frac{du(t)}{dt} &{} \\ \frac{dv(t)}{dt} &{} \\ \frac{d {w(t)}}{dt} &{} \end{bmatrix} = \begin{bmatrix} -\Gamma &{} -\Delta (t) &{} 0 \\ \Delta (t) &{} -\Gamma &{} -\Omega (t) \\ 0 &{} \Omega (t) &{} 0 \end{bmatrix} \begin{bmatrix} u(t) &{} \\ v(t) &{} \\ {w}(t) &{} \end{bmatrix} \end{aligned}$$

Here $\rho _{22}$ and $\rho _{11}$ denotes respectively the atomic populations in the upper state $|2\rangle$ and the ground state $|1\rangle$, *W* is the atomic population inversion. *u*(*t*) and *v*(*t*) are the real and imaginary part of the off-diagonal element $2 \rho _{12}(t)$ (the coherence). For seek of simplicity we introduce the new following variables:

5 $$\begin{aligned} v_{1}(t)= & {} \nu (t)e^{\Gamma t} \end{aligned}$$

6 $$\begin{aligned} u_{1}(t)= & {} u(t)e^{\Gamma t} \end{aligned}$$

7 $$\begin{aligned} {w}_{1}(t)= & {} w(t)e^{\Gamma t}. \end{aligned}$$

By using the change of variables $x=\int \Delta (t)dt$, $g(x)=\frac{\Omega (x)}{\Delta (x)}$ and $h(x)=\frac{\Gamma }{\Delta (x)}$ the Eq. (4) becomes:

8 $$\begin{aligned} \frac{du_{1}}{dx}= & {} -v_{1}(x) \end{aligned}$$

9 $$\begin{aligned} \frac{dv_{1}}{dx}= & {} u_{1}(x)-g(x){w}_{1}(x) \end{aligned}$$

10 $$\begin{aligned} \frac{d {w}_{1}}{dx}= & {} h(x){w}_{1}(x)+g(x)v_{1}(x). \end{aligned}$$

In order to obtain the exact solution of the Bloch equations, we perform repeated differentiation and substitution of Eqs. (8), (9) and (10). Hence, this leads to a linear third-order ordinary differential equation:

11 $$\begin{aligned} \frac{d^3u_{1}}{dx^3}+\frac{\alpha ^2}{x}\frac{du_{1}}{dx}=0. \end{aligned}$$

By solving the last equation we get $u_{1}(t)$, $v_{1}(t)$ and $w_{1}(t)$ in terms of the Bessel functions (type 1 and 2). Next we consider the case $u(0) = 0,\; v(0) = 0,\; w(0) = -1$ where $x(0)=\frac{\alpha ^2}{1-e^{\alpha ^2}}$, the exact solutions of the real and imaginary part of the coherence as well as the population inversion are obtained (see the Supplementary Equations and Theoretical Method in the Supplementary Materials). This corresponds to the case where the population is initially in the ground state. The atomic population inversion at t $\rightarrow$ $\infty$ is given by:

12 $$\begin{aligned} w(\infty )= \frac{\bigg (\alpha ^2 e^{\alpha ^2} Y_0(R_\alpha )+Y_1(2\alpha ^2)(1+e^{\alpha ^2})\bigg ) J_1(R_\alpha )-\bigg (\alpha ^2 e^{\alpha ^2} J_0(R_\alpha )+J_1(2\alpha ^2)(1+e^{\alpha ^2})\bigg )Y_1(R_\alpha )}{\alpha ^2 (1+e^{\alpha ^2})\bigg (J_0(R_\alpha ) Y_1(R_\alpha )- J_1(R_\alpha )Y_0(R_\alpha )\bigg )}. \end{aligned}$$

The explicit expressions of $u_1(x)$, $v_1(x)$, $c_1$, $c_2$, $c_3$, *u*(*t*), *v*(*t*) and *w*(*t*) are given in the Supplementary Materials by the Supplementary Eqs. (S1–S7), respectively.

## Discussion

This section discusses the effect of a q-deformed shaped laser on the dynamic of the atomic population inversion, the real and the imaginary parts of the coherence where the analysis is performed in a realistic scenario at the resonance and off-resonance.

**Figure 2:**
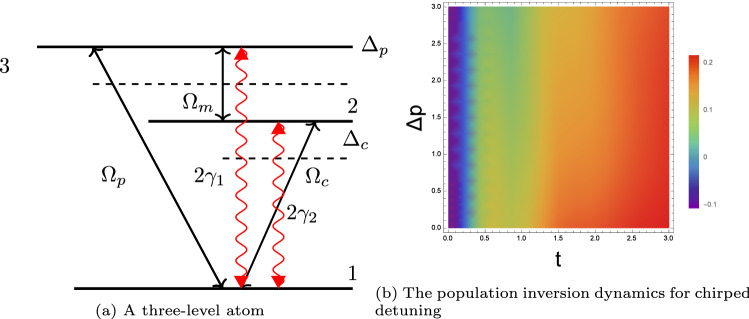
(**a**) A three-level atom excited by a q-deformed control laser and a weak probe field, (**b**) The temporal dynamics of the atomic population inversion for various detunings.

Therefore, we consider an optically dense atomic medium in a three level configuration where, a weak microwave field couples the level pairs $|3\rangle$ to $|2\rangle$ and a strong q-deformed waveform couples the ground state $|1\rangle$ to the upper state $|2\rangle$, (see Fig. 2a).

This configuration can be experimentally realized in $Rb^{87}$ ensemble with $5S_{1/2}$, F = 1, m = 1, $5P_{1/2}$, F = 1, m = 0 and $5S_{1/2}$, F = 2, m = 1^36^. $\gamma _{1,2}$ are the radiative decay rates from higher levels $|3\rangle$ to $|1\rangle$ and $|2\rangle$ to $|1\rangle$. In addition, the detunings of the probe and the control field are denoted by $\Delta _{p}=\omega _{31}-\omega _{p}$ and $\Delta _{c}=\omega _{21}-\omega _{c}$, respectively.

**Figure 3:**
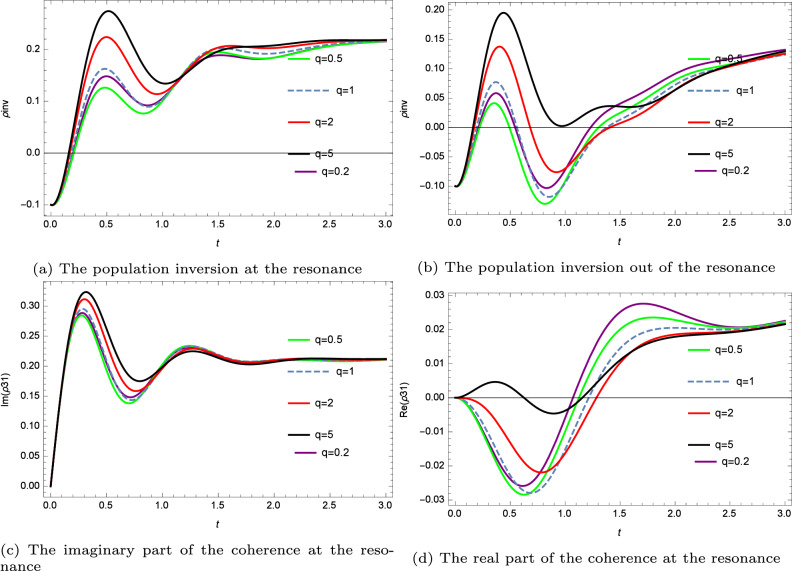
$Rb^{87}$ in a three level configuration controlled by various q-deformed waveforms: the atomic population inversion and the coherence in terms of time t scaled to the pulse rise time.

**Figure 4:**
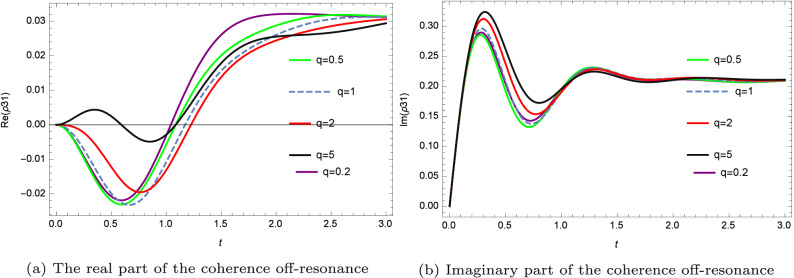
The real and the imaginary part of the coherence off-resonance and for various deformations.

In this case, the Hamiltonian is given by:

13 $$\begin{aligned} H=\Delta _{p}|3\rangle \langle 3|+\Delta _{c}|2\rangle \langle 2|-(\Omega _{c}|2\rangle \langle 1|+\Omega _{c}^{*} |1\rangle \langle 2|+\Omega _{p}|3\rangle \langle 1|+\Omega _{m}|3\rangle \langle 2|) \end{aligned}$$

hence, the temporal evolution of the system is governed by the 3 $\times$ 3 density matrix equations given in the Supplementary Materials by the Density Matrix with the set of equations (Supplementary Eq. S15). By solving the density matrix, we report in (Fig. 2b) the atomic population inversion for a q-deformed control laser and variable detuning while we plot in (Fig. 3a,b) the dynamic of the atomic population inversion for various deformations of the field. To get a deeper insight into the dependence of the probe field’s absorption and dispersion properties on the q deformation of the control field, we plot Im($\rho _{31}$) and Re($\rho _{31}$) (see Figs. 3c,d and 4). All the calculations are performed by considering the initial populations $\rho _{22}=0.2$ and $\rho _{33}=0.1$, respectively. Furthermore, at the resonance, $\Delta _{p}=\Delta _{c}=0.2$, whereas at the off-resonance, $\Delta _{p}=0.2$, $\Delta _{c}=0.7$.

In Figs. 3a,b, we notice that although state $|3\rangle$ is initially less populated than state $|2\rangle$, this pulse achieves a quick population inversion (t is in units of $\tau$, and $\tau$ is scaled to the pulse rise time). In the off-resonance case, the population dynamics presents a similar behaviour as the resonance case, nevertheless the attained maximum amplitude and the stationary value of the dynamics of the population inversion are reduced. In addition, we note from further simulations not shown here that, for very large detuning the dynamics of the atomic population inversion are barely sensitive to the asymmetry parameter q.

Figure 3b,c show that the dynamics of the real and imaginary parts of the coherence for the resonant case depend on the asymmetry of the pulse. In fact, the maximum of the real part of the coherence is attained for a small q factor ( at t = 2.5) while the rise of the q-parameter enhances the imaginary part of the coherence.

It is worth noting that the q-deformation is interpreted in terms of the asymmetry (since the q is a scaling parameter of the asymmetry in the system). Thus, the system’s dynamic is controlled by adjusting the scaling asymmetry factor q of the q-deformed pulse (for instance, we achieve the highest atomic population inversion at the resonance and for a high value of the q-deformation). As observed in Figs. 3 and 4, the stationary values of coherence and population inversion are independent of asymmetry, although they are affected by the general pulse shape family.

Therefore, we show that with an initial non-inverted population, a significant stationary population inversion (about 20$\%$ for the resonant case) is achieved. This result is important for quantum information processing and the implementation of the quantum memory. As previously stated, increasing the q-asymmetry factor increases the maximum of the population inversion while having no effect on its stationary value. By pumping with a periodic pulse train, we expect to maintain the high value of the population inversion.

Finally, it is worth noting that such an atomic system is not only controllable via the strong pulse, but it is also susceptible to the action of the microwave field. Indeed, it has been found that^37^ a slight change in the strength of the microwave field generates a remarkable impact on the transmitted probe field. This is due to the fact that the coherence between states $|2\rangle$ and $|3\rangle$ can change significantly by tuning the microwave field.

## Conclusion

In this work we suggest an analytical method to solve the Bloch equations that describe a two-level atom driven by a q-deformed shaped laser. Hence, the exact solutions of the atomic population inversion as well as the coherence are obtained. In addition, we analyse a realistic scenario described by an optically dense atomic ensemble, where the a q-deformed shaped laser controls the Stokes transition. We analyse the effect of the q-deformation on the dynamics of the atomic population inversion as well as the real and the imaginary parts of the coherence. We demonstrate that, the atomic system dynamics can be fully controlled through the manipulation of the asymmetry scaling parameter q.

## Supplementary Information

Supplementary Information.

## Data Availability

The datasets used in this study are available upon reasonable request from the corresponding author.
