# Supplementary material for: Analysis of a q-deformed hyperbolic short laser pulse in a multi-level atomic system
Source: Sci Rep. 2022 Jun 3;12:9308. doi: 10.1038/s41598-022-13407-7 (PMC9166771; doi:10.1038/s41598-022-13407-7)
Supplement: Supplementary file 1 — Supplementary Information. [file 41598_2022_13407_MOESM1_ESM.pdf]

Supplementary Materials: Analysis of a Q-Deformed Hyperbolic Short Laser Pulse in a Multi-Level Atomic System.

N. Boutabba[1,*], S. Grira[2] and H. Eleuch[3,4]

[1] *Fatima College of Health Sciences, Institute of Applied Technology, Abu Dhabi, UAE.*

[2] *Department of Applied Sciences and Mathematics, Abu Dhabi University, Abu Dhabi, UAE.*

[3] *Department of Applied Physics and Astronomy, University of Sharjah, Sharjah, UAE.*

[4] *Institute for Quantum Science and Engineering, Texas A&M University, College Station, TX 77843, USA.*

Abstract

A fast laser pulse with a q-deformed hyperbolic function shape is investigated in a Multi-level atomic system. Therefore, we first derive the exact solution of the Bloch equations describing a two-level atom excited by a q-deformed laser pulse with dephasing and time-dependent detuning. Next, we analyze the atomic population inversion at resonance and off-resonance of a Rubidium 87 three-level atom driven by a classical weak field and a strong q-deformed control laser. Finally, in order to get a deeper insight of the probe field's absorption and dispersion properties, we investigate the coherence's dependence on the q-deformation. Our work demonstrates that, the atomic system can be fully controlled through the manipulation of the asymmetry scaling parameter q of the q-deformed hyperbolic laser wave-form.

Keywords: Laser shaping, Multi-level atom, Quantum control

Content

Supplementary Equations and Theoretical Method

Supplementary Density Matrix

Supplementary Equations and Theoretical Method

First, we consider a two level atom driven by a q-deformed pulse (Eq.2 in the main manuscript) with chirped frequencies (Eq.3 in the main manuscript). The Hamiltonian of the system is given by $H = \Delta(t)\sigma_x\frac{\hbar}{2} + \Omega(t)\sigma_z\frac{\hbar}{2})$. Next, we write the master equation of the Hermitian density matrix as: $\frac{d\rho(t)}{dt} = -i\left[H,\rho\right] + \frac{\Gamma}{2}(\sigma_z\rho\sigma_z - \rho)$. Then, we derive the Bloch equations (see Eq.(4) in the main manuscript) where the variables u(t)and v(t) are respectively the real and imaginary parts of the coherence ρ_{12} and w(t) is the atomic population inversion.

By introducing the new variables $u_1(t)$, $v_1(t)$ and $w_1(t)$ (see Eq.(4), Eq.(5) and Eq.(6) in the main manuscript) in addition to the change of variables x and g, we get a new set of equations (see Eq.(8), Eq.(9) and Eq.(10) in the main manuscript). By repeated differentiations and substitutions we derive a new linear third order differential equation. Solving Eq.(11)(presented in the main manuscript) gives the exact solutions of $u_1(t)$, $v_1(t)$ and $w_1(t)$ in terms of the Bessel equations (type 1 and 2) as follows:

$$u_1(x) = C_1 + C_2 x J_2(2\alpha\sqrt{x}) + 3C_3 x Y_2(2\alpha\sqrt{x}) \tag{S1}$$

$$v_1(x) = -C_2\alpha\sqrt{x}\bigg(J_1(2\alpha\sqrt{x}) + C_3 Y_1(2\alpha\sqrt{x})\bigg) \tag{S2}$$

as mentioned above, the populations are considered initially to be in the ground state: $u(0) = 0,\ v(0) = 0,\ w(0) = -1$ where $x(0) = \frac{\alpha^2}{1-e^{\alpha^2}}$, hence we have:

$$C_1 = \frac{-1}{1+e^{-\alpha^2}} \tag{S3}$$

$$C_2 = \frac{Y_1\left(\frac{2\alpha^2}{\sqrt{1+e^{-\alpha^2}}}\right)}{\left(J_1\left(\frac{2\alpha^2}{\sqrt{1+e^{-\alpha^2}}}\right)Y_0\left(\frac{2\alpha^2}{\sqrt{1+e^{-\alpha^2}}}\right) - J_0\left(\frac{2\alpha^2}{\sqrt{1+e^{-\alpha^2}}}\right)Y_1\left(\frac{2\alpha^2}{\sqrt{1+e^{-\alpha^2}}}\right)\right)\alpha^2} \tag{S4}$$

$$C_3 = \frac{J_1\left(\frac{2\alpha^2}{\sqrt{1+e^{-\alpha^2}}}\right)}{\left(J_0\left(\frac{2\alpha^2}{\sqrt{1+e^{-\alpha^2}}}\right)Y_1\left(\frac{2\alpha^2}{\sqrt{1+e^{-\alpha^2}}}\right) - J_1\left(\frac{2\alpha^2}{\sqrt{1+e^{-\alpha^2}}}\right)Y_0\left(\frac{2\alpha^2}{\sqrt{1+e^{-\alpha^2}}}\right)\right)\alpha^2} \tag{S5}$$

Finally, the exact solutions of $u(t)$, $v(t)$ and $w(t)$ are:

$$u(t) = \left[\frac{-1}{1+e^{-\alpha^2}} + \frac{Y_1\left(\frac{2\alpha^2}{\sqrt{1+e^{-\alpha^2}}}\right)J_2\left(2\alpha\sqrt{\frac{\alpha^2}{1+e^{-\alpha^2}e^{-2t}}}\right)}{\left(1+e^{-\alpha^2}e^{-2t}\right)\left(J_1\left(\frac{2\alpha^2}{\sqrt{1+e^{-\alpha^2}}}\right)Y_0\left(\frac{2\alpha^2}{\sqrt{1+e^{-\alpha^2}}}\right) - J_0\left(\frac{2\alpha^2}{\sqrt{1+e^{-\alpha^2}}}\right)Y_1\left(\frac{2\alpha^2}{\sqrt{1+e^{-\alpha^2}}}\right)\right)} + \right. \tag{1}$$

$$\left. \frac{J_1\left(\frac{2\alpha^2}{\sqrt{1+e^{-\alpha^2}}}\right)Y_2\left(2\alpha\sqrt{\frac{\alpha^2}{1+e^{-\alpha^2}e^{-2t}}}\right)}{\left(1+e^{-\alpha^2}e^{-2t}\right)\left(J_0\left(\frac{2\alpha^2}{\sqrt{1+e^{-\alpha^2}}}\right)Y_1\left(\frac{2\alpha^2}{\sqrt{1+e^{-\alpha^2}}}\right) - J_1\left(\frac{2\alpha^2}{\sqrt{1+e^{-\alpha^2}}}\right)Y_0\left(\frac{2\alpha^2}{\sqrt{1+e^{-\alpha^2}}}\right)\right)}\right]e^{-t} \tag{S6}$$

$$v(t) = \alpha\sqrt{\frac{\alpha^2}{1+e^{-\alpha^2}e^{-2t}}}\left[\frac{Y_1\left(\frac{2\alpha^2}{\sqrt{1+e^{-\alpha^2}}}\right)J_1\left(2\alpha\sqrt{\frac{\alpha^2}{1+e^{-\alpha^2}e^{-2t}}}\right)}{\left(J_1\left(\frac{2\alpha^2}{\sqrt{1+e^{-\alpha^2}}}\right)Y_0\left(\frac{2\alpha^2}{\sqrt{1+e^{-\alpha^2}}}\right) - J_0\left(\frac{2\alpha^2}{\sqrt{1+e^{-\alpha^2}}}\right)Y_1\left(\frac{2\alpha^2}{\sqrt{1+e^{-\alpha^2}}}\right)\right)} + \right.$$
$$\left. \frac{J_1\left(\frac{2\alpha^2}{\sqrt{1+e^{-\alpha^2}}}\right)Y_1\left(2\alpha\sqrt{\frac{\alpha^2}{1+e^{-\alpha^2}e^{-2t}}}\right)}{\left(J_0\left(\frac{2\alpha^2}{\sqrt{1+e^{-\alpha^2}}}\right)Y_1\left(\frac{2\alpha^2}{\sqrt{1+e^{-\alpha^2}}}\right) - J_1\left(\frac{2\alpha^2}{\sqrt{1+e^{-\alpha^2}}}\right)Y_0\left(\frac{2\alpha^2}{\sqrt{1+e^{-\alpha^2}}}\right)\right)}\right]e^{-t} \tag{S7}$$

then, we define the following positive constants in terms of α :

$$R_\alpha = \frac{2\alpha^2}{\sqrt{1+e^{-\alpha^2}}} \tag{S8}$$
$$K_\alpha = \frac{2\alpha^2 e^{1+\frac{\alpha^2}{2}}}{\sqrt{1+e^{\alpha^2+2t}}} \tag{S9}$$
$$T_\alpha = e^{2t} + e^{-\alpha^2} + e^{\alpha^2+2t} + 1 \tag{S10}$$
$$G_\alpha = e^{\frac{-3\alpha^2}{2}-t} + e^{\frac{-\alpha^2}{2}-t} \tag{S11}$$
$$H_\alpha = \sqrt{1+e^{\alpha^2+2t}}\alpha^2(1+e^{-\alpha^2-2t})(1+e^{\alpha^2}) \tag{S12}$$

This leads to the atomic population inversion in terms of α as follows:

$$\begin{aligned} w(t) = \quad & -\frac{1}{H_\alpha(J_0(R_\alpha)Y_1(R_\alpha) - J_1(R_\alpha)Y_0(R_\alpha))}\left[e^{\frac{\alpha^2}{2}-t}\left(Y_1(R_\alpha)T_\alpha J_1(K_\alpha) - \right.\right. \\ & J_1(R_\alpha)T_\alpha Y_1(K_\alpha) + Y_1(G_\alpha)T_\alpha J_0(K_\alpha) - J_1(G_\alpha)T_\alpha Y_0(K_\alpha) + \\ & \left.\left.\left(J_0(R_\alpha)Y_1(R_\alpha) - J_1(R_\alpha)Y_0(R_\alpha)\right)(e^{\frac{\alpha^2}{2}+t} + e^{-\frac{\alpha^2}{2}-t})\right)\right] \end{aligned} \tag{S13}$$

and

$$w(\infty) = \frac{(\alpha^2 e^{\alpha^2} Y_0(R_\alpha) + Y_1(2\alpha^2)(1+e^{\alpha^2}))J_1(R_\alpha) - (\alpha^2 e^{\alpha^2} J_0(R_\alpha) + J_1(2\alpha^2)(1+e^{\alpha^2}))Y_1(R_\alpha)}{\alpha^2(1+e^{\alpha^2})(J_0(R_\alpha)Y_1(R_\alpha) - J_1(R_\alpha)Y_0(R_\alpha))} \tag{S14}$$

Supplementary Density Matrix

The analysis of the three level atom (see the discussion in the main manuscript) was performed numerically using the temporal evolution of the density matrix elements given by:

$$\begin{aligned}
\frac{d}{dt}\rho_{22} &= -2\gamma_2\rho_{22} + i\Omega_c(\rho_{12} - \rho_{21}) + i\Omega_m(\rho_{32} - \rho_{23}) \\
\frac{d}{dt}\rho_{33} &= -2\gamma_1\rho_{33} + i\Omega_p(\rho_{13} - \rho_{31}) + i\Omega_m(\rho_{23} - \rho_{32}) \\
\frac{d}{dt}\varrho_{21} &= -(\gamma_2 + i\Delta_c)\rho_{21} + i\Omega_c(\rho_{11} - \rho_{22}) - i\Omega_p\rho_{23} + i\Omega_m\rho_{31} \\
\frac{d}{dt}\varrho_{31} &= -(\gamma_1 + i\Delta_p)\rho_{31} + i\Omega_p(\rho_{11} - \rho_{33}) - i\Omega_c\rho_{32} + i\Omega_m\rho_{21} \\
\frac{d}{dt}\varrho_{32} &= -(\gamma_1 + \gamma_2 - i\Delta_c + i\Delta_p)\rho_{32} + i\Omega_p\rho_{12} - i\Omega_c\rho_{31} + i\Omega_m(\rho_{22} - \rho_{33})
\end{aligned} \tag{S15}$$
